# Effectiveness of long K-wire percutaneous intramedullary fixation for distal radius metaphyseal-diaphyseal transition zone fractures

**DOI:** 10.3389/fped.2026.1741641

**Published:** 2026-02-19

**Authors:** Wenjie Gao, Feiyang Zhu, Rui Wang, Dhruvkumar Arvindbhai Vasoya, Feng Yao, Fuyong Zhang, Yunfang Zhen, Xiaodong Wang

**Affiliations:** Department of Pediatric Orthopedics, Children’s Hospital of Soochow University, Suzhou, China

**Keywords:** children, distal radius, metaphyseal-diaphyseal transition zone, fracture, long K-wire, percutaneous, intramedullary

## Abstract

**Aims:**

Distal radius metaphyseal-diaphyseal transition zone fractures in children are challenging due to their distinct anatomical features and slower healing speed. Although both percutaneous long Kirschner wire (K-wire) intramedullary fixation and open reduction with plate and screw fixation are commonly employed, there is no clear consensus on the optimal surgical strategies. This study aimed to compare the clinical outcomes of percutaneous long K-wire intramedullary fixation and open reduction with plate and screw fixation.

**Methods:**

We conducted an analysis of pediatric patients aged 8–14 years treated for distal radius metaphyseal-diaphyseal transition fractures between August 2021 and July 2023. Patients were stratified into two cohorts: the Long K-wire group, treated via closed reduction and percutaneous intramedullary fixation targeting the radial medullary isthmus, and the Plate group, treated via open reduction and internal fixation (ORIF). Perioperative metrics (operative time, incision length, hospital stay), functional outcomes (Gartland-Werley score), radiographic parameters, and complication rates were compared between the groups.

**Results:**

Both surgical techniques achieved successful fracture union with no significant differences in radiographic alignment or functional recovery; the majority of patients in both groups achieved “Excellent” Gartland-Werley score six months after surgery. However, the Long K-wire group demonstrated statistically significant advantages, including shorter operative times, reduced incision lengths, and decreased length of hospital stay (*P* < 0.05). Additionally, the Long K-wire group avoided the need for a second inpatient surgery for hardware removal, which was required for the Plate group. Complication rates, including refracture, were low and comparable between groups.

**Conclusion:**

Percutaneous long K-wire intramedullary fixation is an effective minimally invasive alternative in selected patients to plate fixation for treating distal radius metaphyseal-diaphyseal transition fractures. It offers minimal surgical trauma, accelerates recovery, and lowers risk of complications while ensuring comparable functional outcomes. Due to these advantages, this technique should be regarded as a clinically useful attempt for pediatric patients.

## 1 Introduction

The distal radius metaphyseal transition is not sufficiently vascularized, and its healing is slower than that of the metaphysis (1). Compared to distal radius fractures, distal radius metaphyseal-diaphyseal transition fractures are associated with a higher risk of complications, such as delayed union, non-union, or displacement. These fractures necessitate distinct treatment approaches compared to typical metaphyseal fractures. However, they do not receive adequate clinical attention.

In 2010, Lieber et al. first proposed the concept of distal metaphyseal transition (DMT) fracture in children (2), Later, many researchers provided a more precise explanation for this fracture, which mainly occurred in the distal third of the metaphyseal transition region of the radius (3). Fractures of the metaphyseal-diaphyseal junction pose considerable challenges (4), with the ongoing debate regarding the most effective surgical approach for distal radial fractures in pediatric patients (5). The primary objective of treatment is to facilitate fracture healing and preserve the normal range of motion in the wrist joints (6, 7). Percutaneous Kirschner wire intramedullary fixation and open reduction with plate and screw internal fixation are the two predominant surgical interventions employed for the treatment of metaphyseal-diaphyseal junction fractures.

This study retrospectively analyzed clinical data from patients with radial metaphyseal-diaphyseal transition fractures treated at our hospital between August 2021 and July 2023. We compared the advantages and disadvantages of closed reduction percutaneous Kirschner wire intramedullary fixation and open reduction plate and screw internal fixation in treating pediatric radial metaphyseal-diaphyseal transition fractures. We also explored the clinical efficacy of percutaneous long Kirschner wire intramedullary fixation for pediatric distal radial metaphyseal-diaphyseal transition fractures.

## 2 Methods

This retrospective study analyzed clinical data from patients with distal radius metaphyseal-diaphyseal transition fractures treated in our hospital between August 1, 2021, and July 31, 2023. Patients were divided into the following groups: percutaneous long Kirschner wire intramedullary (long K-wire group) and open reduction with plates and screws fixation (plate group). General information on the two groups is provided in Table 1.

**Table 1:** Characteristics of the patients at baseline.

Variables	Long K-wire	Steel plate screw	*P* value
	(*n* = 31)	(*n* = 32)	
Age, year	10.26 ± 1.81	11.88 ± 1.66	<0.01
Male, *n* (%)	28 (87.5%)	32 (100%)	0.113
Injured Side, *n* (%)			0.098
Left	16 (51.6%)	23 (71.9%)	
Right	15 (48.4%)	9 (28.1%)	
Dominant Hand, *n* (%)			0.098
Yes	15 (48.4%)	9 (28.1%)	
No	16 (51.6%)	23 (71.9%)	

Information of the patients: There was a statistical difference in age between the two groups, but no statistical difference in gender, injured Side and dominant hand.

### 2.1 Inclusion criteria and exclusion criteria

#### 2.1.1 Inclusion criteria

Closed distal radius metaphyseal-diaphyseal transition fractures;
Fresh fractures (≤7 days);
Patients aged between 8 and 14 years;
Surgical treatment with either percutaneous long K-wire intramedullary fixation or open reduction with plate and screw fixation, supplemented with tubular plaster or splint after surgery.

#### 2.1.2 Exclusion criteria

K-wire shorter than 7 cm;
Previous limb surgery;
Fractures lasting 7 days, pathological fractures associated with bone cyst, osteochondroma;
Open fractures with bone exposure or soft tissue damage;
Complex fractures with wrist and forearm deformities;
Treatment with other surgical techniques, such as elastic intramedullary nailing;
Conservative treatment, such as cast immobilization only;
Patients with systemic diseases or significant neurovascular injuries;
Incomplete clinical data, including essential x-rays or key clinical data.

### 2.2 Operations

**Figure 1:**
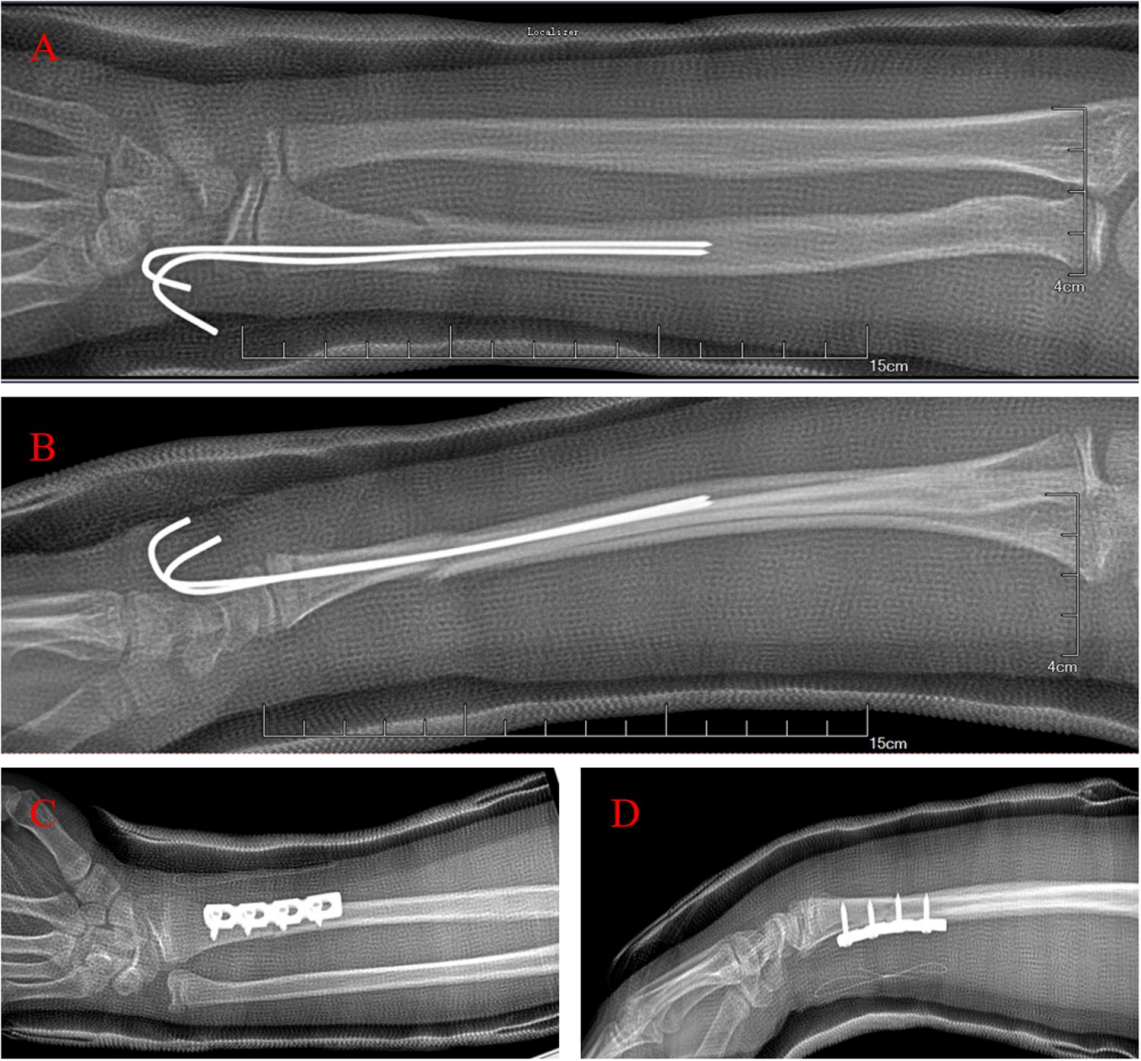
Operation methods. **(A,B)** Long Kirschner wire group. **(C,D)** Plate and screw group.

Group 1 underwent percutaneous long Kirschner wire intramedullary fixation (Long K-wire group, Figures 1A,B). The patient was positioned supine under general anesthesia, and the injured limbs was prepared under sterile conditions. The operator and assistant held the distal and proximal ends of the fracture, respectively, applying anti-traction, flexion, and reduction of the fracture end to the volar or dorsal side. The C-arm machine showed that the fracture was well-aligned. A Kirschner wire was employed to fix the fracture end and reach the bone marrow cavity of the proximal end of the fracture. An additional K-wire was inserted for additional stabilization, crossing the first K-wire into the bone marrow cavity or bone cortex of the proximal end of the fracture. In additional fluoroscopy, the fracture exhibited proper alignment, the K-wire was in place, and the fixation was firm. The K-wire end was shortened and bent, and the sterile gauze material was bandaged and supplemented with plaster external fixation.

Group 2 underwent open reduction and internal fixation with a plate and screws (Plate Group, Figures 1C,D). In this group, the patient was positioned supine under general anesthesia and the injured limbs was prepared under sterile conditions. A suitable incision was made at the fracture site, and the skin and fascia were dissected. Blood vessels were separated and protected, and the fracture end was exposed through the intermuscular space. A metal osteosynthesis plate was placed after direct vision reduction, and fixation was achieved with 4–6 screws. The C-arm revealed that the fracture fixation was and the fixation appeared stable and the fragments were adequately aligned. The wound was cleaned with normal saline, and sutured in layers, and a sterile dressing was applied with external plaster support.

### 2.3 Statistical analysis

Statistical analysis was conducted using *SPSS (version 28.0.1.1*). Measurement data (radiographic evaluation, incision length, operation duration, postoperative hospital stay, forearm function, and complications) are expressed as $\bar{x}\pm s$. Independent samples *t* test was employed to compare the two groups. Count data were compared using $x^{2}$ test or corrected $x^{2}$ test. *P* < 0.05 was considered statistically significant.

### 2.4 Outcomes and measures

#### 2.4.1 Perioperative indexes

Operation length duration was recorded from the start of the procedure (after anesthesia) to the completion of plaster fixation.
Postoperative hospital stay was measured from the first postoperative day to discharge, excluding the day of surgery.
The main complications included wound infection, non-union, loss of fracture reduction, unplanned secondary surgery.

#### 2.4.2 Clinical evaluation index

Incision length: In the long Kirschner wire group, the incision length was calculated based on the total diameter of the Kirschner wire. In the plate and screws group, the incision length was calculated based on the surgical incision in the operation record.
Forearm function: The Gartland-Werley score (Table 2) was utilized to measure forearm function.

The score is based on a list of items categorised into four domains namely:

Residual deformity, subjective evaluation, objective evaluation, and complications.

The list of items with relative scores and cut-offs is presented below:

**Table 2:** The Gartland-Werlay Score.

Section	Results	Points
1	Residual deformities	
1.1	Prominent ulnar styloid	1
1.2	Residual dorsal tilt	2
1.3	Radial deviation of hand	2–3
2	Subjective evaluation	
2.1	No pain, disability or limitation in motion (Excellent)	0
2.2	Occasional pain, slight limitation in motion, no disability (Good)	2
2.3	Occasional pain, some limitation of motion, feeling of weakness in the wrist, no particular disability if careful, activities slightly restricted (Fair).	4
2.4	Pain, limitation of motion, disability, activities more or less markedly restricted (Poor)	6
3	Objective evaluation	
3.1	Loss of dorsiflexion	5
3.2	Loss of ulnar deviation	3
3.3	Loss of supination	2
3.4	Loss of palmar flexion	1
3.5	Loss of radial deviation	1
3.6	Loss of circumduction	1
3.7	Pain in distal radio-ulnar joint	1
4	Complications (Arthritic change)	
4.1	Minimal	1
4.2	Minimal with pain	3
4.3	Moderate	2
4.4	Moderate with pain	4
4.5	Severe	3
4.6	Severe with pain	5
4.7	Nerve complications (Median)	1–3
4.8	Poor finger function due to cast	1–2

Cut-off scores for end-results point ranges are presented as following:

0–2: Excellent
3–8: Good
9–20: Fair
21 or above: Poor

(This rating form is excerpted from www.physio-pedia.com/)

#### 2.4.3 Radiographic evaluation index

**Figure 2:**
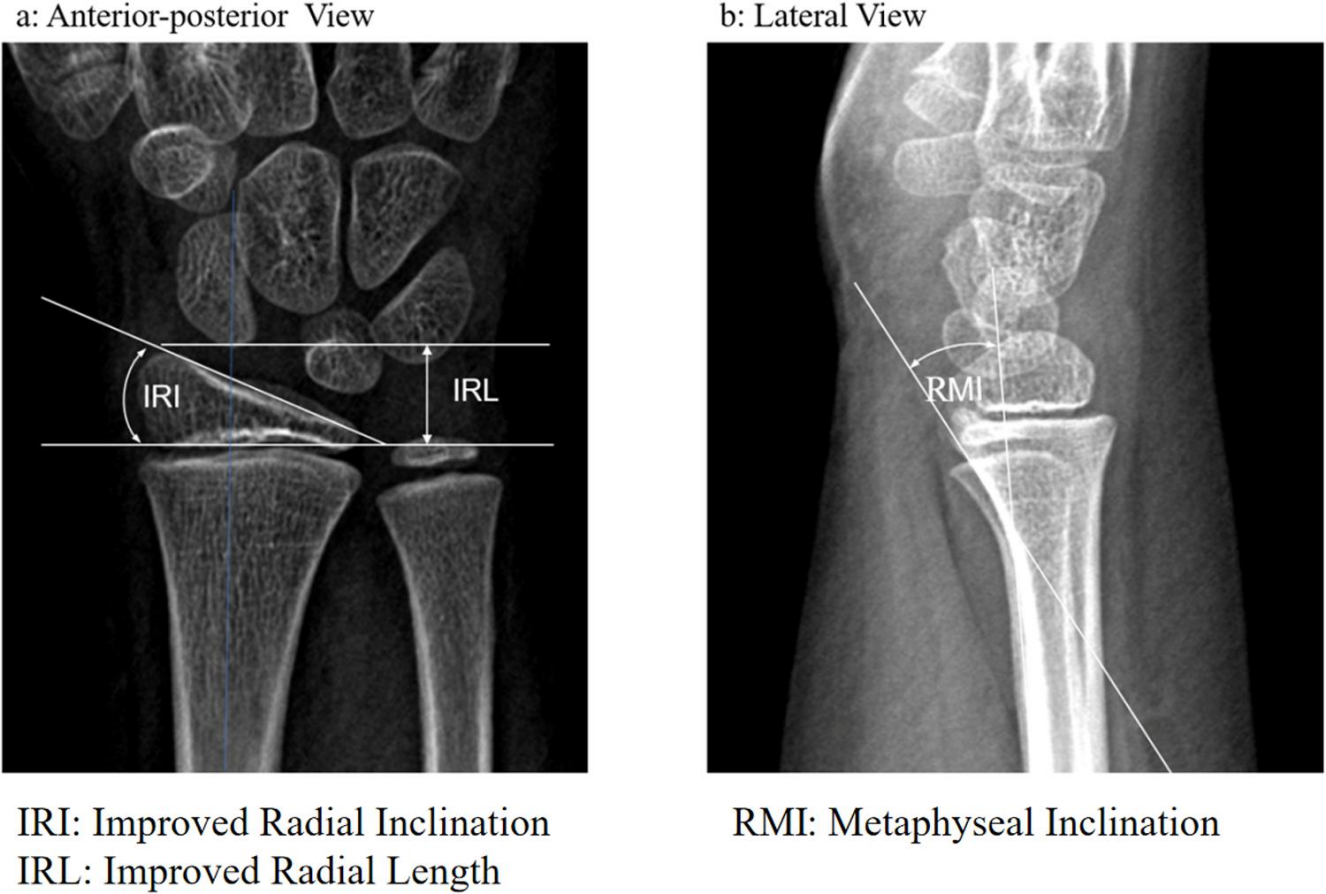
Measurement method. **(a)** IRI and IRL were measured in the A-P view. Improved radial inclination (IRI): the ulnar deviation angle of the immature radius in children; Improved radial length (IRL): the radial length of the immature radius in children. **(b)** RMI was measured in the lateral view. Radius metaphyseal inclination (RMI): the angle between radial metaphysis and radial shaft.

Radiographic Evaluation (Figure 2): Radius metaphyseal inclination (RMI), improved radial inclination (IRI), and improved radial length (IRL) were measured based on x-ray images. RMI is defined as the angle between the radial metaphyseal and radial shaft. IRI refers to the ulnar deviation angle of the immature radius of children, which is different from the radial inclination, and especially assesses the radius of children. IRL refers to the radial length of the immature radius of children and evaluates the radial length of children.

## 3 Results

### 3.1 Perioperative data

Surgery was successfully completed in all cases. In the long K-wire group, four participants were treated with a single long K-wire percutaneous intramedullary fixation, one participant was treated with 3 K-wires for stable fixation. The long K-wire group had a significantly shorter operative time and postoperative hospital stays compared to the plate group (*P* < 0.05) (Table 3).

**Table 3:** Perioperative results.

Variables	Long K-wire	Steel plate screw	*P* value
	(*n* = 31)	(*n* = 32)	
Operation duration, min	46.00 ± 18.02	72.34 ± 24.32	<0.001
Postoperative hospital stay, day	2.29 ± 0.82	3.66 ± 1.15	<0.001
Surgical Wound, cm	0.32 (0.32, 0.35)	5.50 (5.00, 7.00)	<0.001

Perioperative data: There was no statistical difference in operation duration, postoperative hospital stays and Surgical Wound between the two groups.

### 3.2 Clinical evaluation

The average follow-up time of the two groups was 14 ± 7 months. During the follow-up period, 1 participant in the plate group experienced a refracture due to secondary trauma before plate removal. The refracture was treated with open reduction and replacement of plate internal fixation. Similarly, one participant in the long K-wire group experienced a refracture, which was treated with closed reduction and long K-wire intramedullary fixation. The fractures of both participants healed well after the second surgery. There was no vascular or nerve injury before and after surgery in the two groups. Furthermore, no complications, such as delayed union or non-union occurred during the fracture healing period. The Gartland-Werley score was assessed through telephone follow-up. No residual deformity or arthritis was observed in the plate and screw group. One participant reported limited mobility, along with wrist weakness, reduced dorsiflexion, and impaired palmar flexion. Two other participants in the plate and screw group experienced sustained local pain.

Regarding forearm function measured based on the Gartland-Werley score (Table 4), the results were as follows: Plate Group: 30 excellent and 2 fair. Long K-Wire Group: 30 excellent and 1 fair. There was no significant difference between the two groups (*P* > 0.05, The precise *P*-value is presented in the table).

**Table 4:** Gartland-Werlay score.

Variables	Long K-wire	Steel plate screw	*P* Value
	(*n* = 31)	(*n* = 32)	
Three months after surgery	2.69 ± 0.55	2.82 ± 0.57	0.361
Six months after surgery	1.83 ± 0.38	1.97 ± 0.27	0.096
Last follow-up	1.37 ± 0.12	1.38 ± 0.19	0.804

Clinical Evaluation: There was no statistical difference between the two groups at 3 months, 6 months and the last follow-up.

### 3.3 Radiographic evaluation

The results of image evaluation for both groups are presented in Table 5. Compared to measurements conducted after 1 day and 1 month post-operatively, no significant differences were found between the two groups in terms of RMI, IRI, and IRL (*P* > 0.05).

**Table 5:** Imaging Result.

Variables	Long K-wire	Steel plate screw	*P* value
	(*n* = 31)	(*n* = 32)	
IRI,°			
1 day after surgery	22.35 ± 3.21	22.66 ± 2.09	0.063
1 month after surgery	21.19 ± 2.61	22.78 ± 1.52	0.594
IRL, mm			
1 day after surgery	8.82 ± 1.34	9.49 ± 1.60	0.079
1 month after surgery	9.03 ± 1.23	9.03 ± 1.23	0.089
RMI,°			
1 day after surgery	19.55 ± 4.15	18.75 ± 4.43	0.464
1 month after surgery	20.39 ± 3.37	18.88 ± 5.05	0.167

Radiographic Evaluation: IRI, improved radial inclination; IRL, improved radial length; RMI, radius metaphyseal inclination. There was no statistical difference in IRI, IRL and RMI between the two groups.

## 4 Discussion

### 4.1 Definition of the distal radius metaphyseal-diaphyseal transition zone

**Figure 3:**
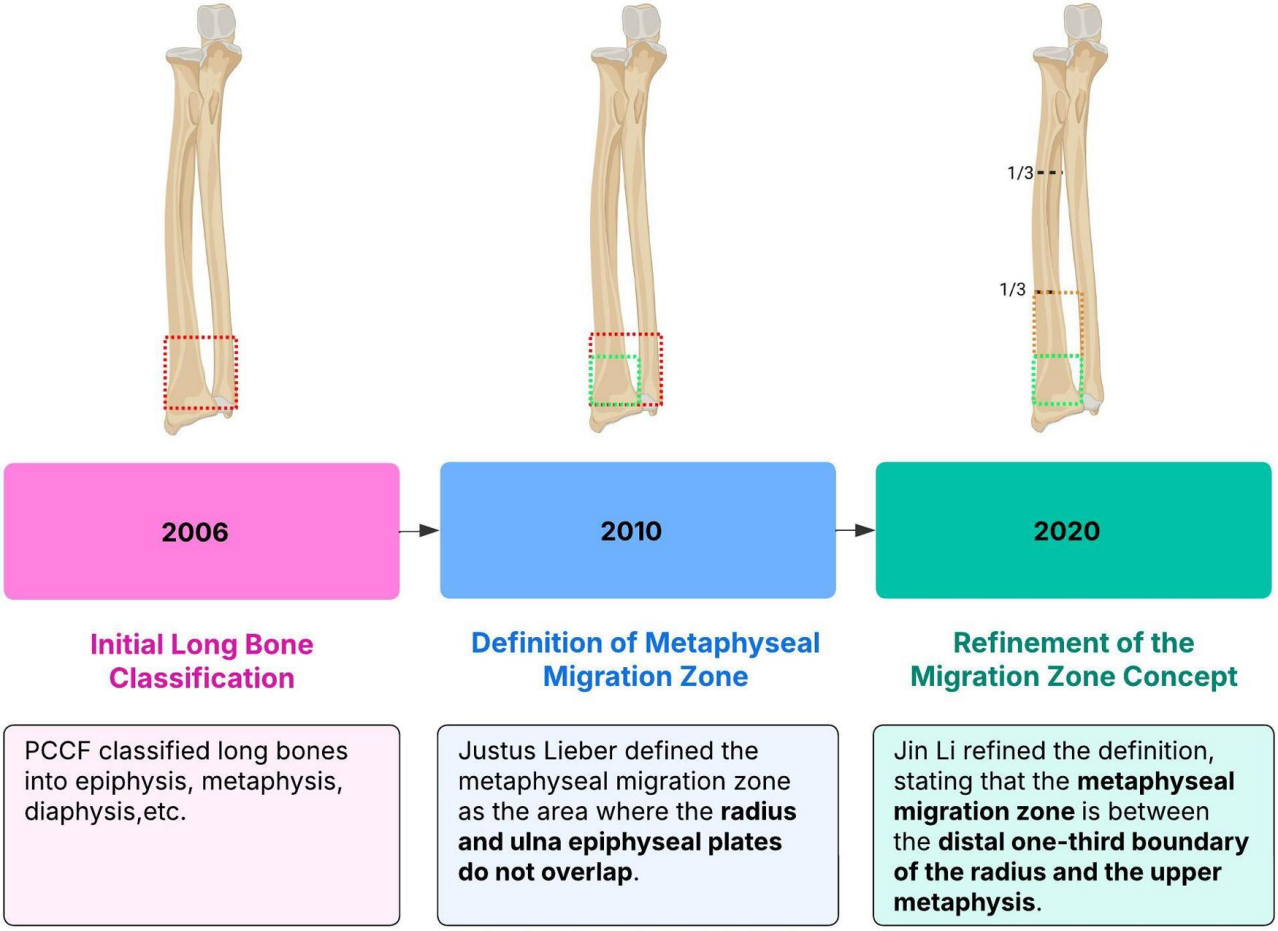
Concept of distal radius metaphyseal-diaphyseal transition zone.

The transition zone was between the distal one-third boundary of the radius and the upper edge of the metaphysis, and this concept has undergone years of development (Figure 3) (8). In 2006, the International Society for the Study of Internal Fixation (PCCF) divided children's long bones into proximal epiphysis and metaphyseal ends, diaphysis, distal epiphysis, and metaphyseal ends (8). Liber et al. took the sum of the widths of the distal epiphyseal plates of the radius and the distal epiphyseal plates of the ulna and radius as the side length. In 2010, they defined the area where the two squares do not overlap as the junction of the metaphyseal plates (2). Ten years later, Li et al. defined the area between the distal one-third boundary of the radius and the upper edge of the metaphysis as the transition zone (9). Distal radius metaphyseal-diaphyseal transition fracture is at high risk of delayed healing and non-healing due to insufficient blood supply. The actual size of the distal radius is affected by several factors, such as the patient's age and height. In this study, distal radius metaphyseal transition referred to the area between the distal one-third boundary of the radius and the upper edge of the metaphysis.

### 4.2 The anatomy

The metaphyseal transition of the distal radius is located in the transition between the metaphysis and the diaphysis, with relatively few vascular perforating branches (10); thus, its local blood supply and remodeling potential are weaker than those of the metaphysis. It is also easily affected by the traction of peripheral tendons such as the extensor pollicis brevis, abductor pollicis longus, and brachioradialis muscle, which elevates the risk of unstable fractures (1).

Fractures observed in children are different from those observed in adults, as the bones contain more organic components, leading to greater toughness and orthopedic ability (11, 12). The generally acceptable angle for complete remodeling of distal radius metaphyseal fractures within 5 years is 30–35 degrees in the sagittal plane and 10 degrees in the coronal plane, but it often differs by age and gender (13). Therefore, appropriate methods that can ensure functional recovery and align with the growth characteristics of children's bones are necessary for treating fractures of the distal radius epiphyseal transition in children (14).

### 4.3 Long Kirschner wire intramedullary fixation

Forearm fractures are among the most common fractures observed. While stable fractures of both adults and children can be managed conservatively through closed reduction and cast immobilization (15), unstable fractures may necessitate surgical fixation (16–19). Open reduction with plate fixation and closed reduction with intramedullary Kirschner wire fixation were two commonly employed surgical methods (20–22), with completely different treatment concepts. One is based on the Arbeitsgemeinschaft für Osteosynthesefragen (AO) principle, offering anatomical reduction and firm fixation. The other one is based on the principles of Biological Osteosynthesis (BO), offering functional reduction with minimal surgical trauma. Most fractures in the distal radial metaphyseal transition zone can be observed among adolescents aged 8–14 years old, when the bone is in the growth stage. Older children (12–14 years old) may require perfect reduction (axial malalignment of less than 10) anatomical reduction and internal fixation based on the AO principle, while younger children (8–10 years old) may be more inclined to choose functional reduction based on the BO principle. This leads to a relative age limitation in the selection of treatment options. Since the growth and development of different individuals are different, the treatment plan cannot be completely determined based on children's age. Through preliminary analysis of the characteristics of radial bone growth, we found that there is a narrow segment in the medullary cavity of the radial shaft. This narrow segment is narrower than the medullary cavity of the proximal and distal ends of the radius and is the narrowest segment of the entire radial medullary cavity. We found that the stability of the Kirschner wire intramedullary fixation greatly increases when the intramedullary Kirschner wire is fixed through this narrow segment. We hypothesize that the intramedullary fixation of the Kirschner wire can increase the stability of the Kirschner wire intramedullary fixation when it is fixed through the narrow segment of the radial medullary cavity (for children aged 8–14 years). Open reduction and internal fixation with plates and screws can achieve anatomical reduction, restore the physiological curvature of the distal radius, and facilitate forearm function recovery in children, but is associated with prospective disadvantage (3). Closed reduction percutaneous Kirschner wire intramedullary fixation technology is minimally invasive (23, 24), promotes the rapid healing of fractures, and requires a short hospitalization period. The surgical approach we advocate causes less trauma compared to plate and screw internal fixation and elastic intramedullary nail fixation. It does not require a second hospitalization for the removal of internal fixation (similar to other percutaneous Kirschner wire internal fixation treatment methods, only the steel pins need to be removed in the outpatient department).

### 4.4 What is long Kirschner wire

In our clinical practice, we noted that long intramedullary Kirschner wires may enhance the stability of Kirschner wire fixation; however, the precise definition of “long” Kirschner wires remains elusive. The optimal length of Kirschner wires for insertion into the medullary cavity of the distal radius is currently under investigation and scrutiny. Therefore, we explored the anatomical features of the radius, which is a key factor in the surgical management of patients with distal radius fractures. Long Kirschner wires enhance the stability of fixation through the medullary cavity because a constricted region exists in the medullary cavity of the central portion of the radial shaft. When a long Kirschner wire extends through the medullary cavity to this constricted region, it significantly improves the stability of fixation through the medullary canal. This empirical conclusion is supported by clinical observations and a profound understanding of the radial isthmus, where the narrowed medullary canal provides a critical biological anchor point for long Kirschner wires to maximize rotational and axial stability (25).

**Figure 4:**
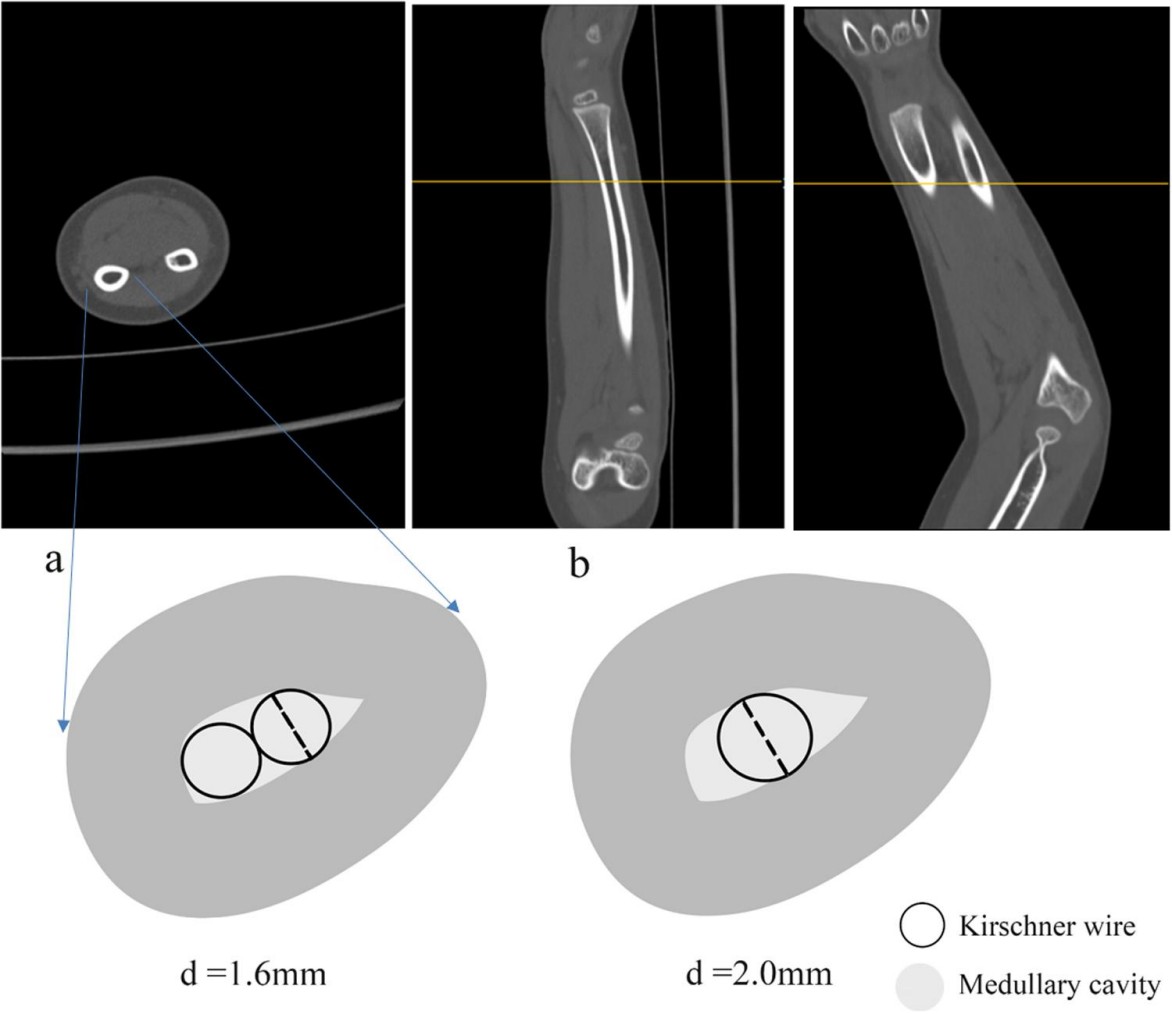
Two strategies for intramedullary fixation with kirschner wires. Two 1. 6 mm-gram K-Wires **(a)** or one 2.0 mm-gram K-Wire **(b)** were fixed in the narrow segment of the pulp cavity.

Based on our experience, intramedullary fixation longer than 7 cm generally suffices to effectively prevent restoration loosening and angulation deformities at the fracture site, so a “long Kirschner wire” denotes a Kirschner wire in the medullary cavity with more than 7 cm length. A narrow segment of the radial medullary cavity appears nearly 7 cm away from the distal end of the radius, and the exact value varies among individuals (Figure 4). Prospective randomized controlled studies with a larger volume of clinical data should validate these findings. Additionally, we recommend the application of forearm tube plaster postoperatively for 3–4 weeks as adjunctive therapy. This method introduces controlled micro-motion at the fracture site, which partly promotes fracture healing by stimulating stress at the fracture site.

### 4.5 Limitations

The incidence of this particular type of fracture is not high, making it difficult to obtain a huge number of participants who meet the requirements for inclusion. Our conclusions require a larger prospective randomized controlled study for confirmation.

## 5 Conclusion

Percutaneous intramedullary fixation with long Kirschner wires is an effective surgical treatment option for treating displaced distal radius metaphyseal-diaphyseal transition fractures.

## Data Availability

The original contributions presented in the study are included in the article/Supplementary Material, further inquiries can be directed to the corresponding author.

## References

[1] Zaidenberg EE Martinez E Zaidenberg CR. Vascularized distal radius bone graft for treatment of ulnar nonunion. J Hand Surg Am. (2018) 43(7):685.81–85. 10.1016/j.jhsa.2018.03.005 29650375

[2] Lieber J Schmid E Schmittenbecher PP. Unstable diametaphyseal forearm fractures: transepiphyseal intramedullary kirschner-wire fixation as a treatment option in children. Eur J Pediatr Surg. (2010) 20(6):395–8. 10.1055/s-0030-1262843 20938899

[3] Wang R Chen D Tang Y Fan M Wang Y Zhuang H A novel method for treating distal radius diaphyseal metaphyseal junction fracture in children. Med Sci Monit. (2023) 29:e939852. 10.12659/MSM.939852 37394785 PMC10327493

[4] Stark D Denzinger M Ebert L Brandl R Knorr C. Therapeutic approaches of diametaphyseal radius fractures in children. Arch Orthop Trauma Surg. (2024) 144(3):1179–88. 10.1007/s00402-023-05118-z 38231205

[5] Liu Y Zhang FY Zhen YF Zhu LQ Guo ZX Wang XD. Treatment choice of complete distal forearm fractures in 8 to 14 years old children. J Pediatr Orthop. (2021) 41(9):e763–7. 10.1097/BPO.0000000000001934 34354028 PMC8439668

[6] Collis JM Mayland EC Wright-St Clair V Signal N. The more I do, the more I can do": perspectives on how performing daily activities and occupations influences recovery after surgical repair of a distal radius fracture. Disabil Rehabil. (2022) 44(19):5440–9. 10.1080/09638288.2021.1936219 34110939

[7] Bredy TM Glasgow C Liddle J Colwell S Holding J Swan S Considering occupational performance during recovery of distal radius fracture: a scoping review. Aust Occup Ther J. (2024) 71(5):798–832. 10.1111/1440-1630.12965 38803065

[8] Slongo T Audige L Schlickewei W Clavert JM Hunter J. International association for pediatric T. Development and validation of the AO pediatric comprehensive classification of long bone fractures by the pediatric expert group of the AO foundation in collaboration with AO clinical investigation and documentation and the international association for pediatric traumatology. J Pediatr Orthop. (2006) 26(1):43–9. 10.1097/01.bpo.0000187989.64021.ml 16439900

[9] Li J Rai S Tang X Ze R Liu R Hong P. Fixation of delayed distal radial fracture involving metaphyseal diaphyseal junction in adolescents: a comparative study of crossed Kirschner-wiring and non-bridging external fixator. BMC Musculoskelet Disord. (2020) 21(1):365. 10.1186/s12891-020-03404-0 32517675 PMC7285434

[10] Jockel CR Zlotolow DA Butler RB Becker EH. Extensile surgical exposures of the radius: a comparative anatomic study. J Hand Surg Am. (2013) 38(4):745–52. 10.1016/j.jhsa.2012.12.029 23419709

[11] Houshian S Holst AK Larsen MS Torfing T. Remodeling of salter-harris type II epiphyseal plate injury of the distal radius. J Pediatr Orthop. (2004) 24(5):472–6. 10.1097/01241398-200409000-00004 15308894

[12] Greig D Silva M. Management of distal radius fractures in adolescent patients. J Pediatr Orthop. (2021) 41(Suppl 1):S1–5. 10.1097/BPO.0000000000001778 34096530 PMC10364841

[13] Wilkins KE. Principles of fracture remodeling in children. Injury. (2005) 36(Suppl 1):A3–11. 10.1016/j.injury.2004.12.007 15652934

[14] Kahraman HC Sibar K Alemdaroglu KB Subasi IO Gokgoz MB. Is three-point index reliable in the follow-up of the distal radius metaphys fractures in the pediatric age group? J Pediatr Orthop B. (2023) 32(4):369–77. 10.1097/BPB.0000000000001032 36377954

[15] Ma H Ruan B Li J Zhang J Wu C Tian H Topology-Optimized splints vs casts for distal radius fractures: a randomized clinical trial. JAMA Netw Open. (2024) 7(2):e2354359. 10.1001/jamanetworkopen.2023.54359 38306099 PMC10837751

[16] Sundermann B Mörs K Frank J Marzi I. Unterarmfrakturen und distale radiusfrakturen bei kindern. Op-Journal. (2018) 34(03):290–301. 10.1055/a-0623-7069

[17] Krukhaug Y Ugland S Lie SA Hove LM. External fixation of fractures of the distal radius: a randomized comparison of the hoffman compact II non-bridging fixator and the dynawrist fixator in 75 patients followed for 1 year. Acta Orthop. (2009) 80(1):104–8. 10.1080/17453670902807433 19234890 PMC2823232

[18] Sinikumpu JJ Nietosvaara Y. Treatment of distal forearm fractures in children. Scand J Surg. (2021) 110(2):276–80. 10.1177/1457496920983104 33372581

[19] Laaksonen T Kosola J Nietosvaara N Puhakka J Nietosvaara Y Stenroos A. Epidemiology, treatment, and treatment quality of overriding distal metaphyseal radial fractures in children and adolescents. J Bone Joint Surg Am. (2022) 104(3):207–14. 10.2106/JBJS.21.00850 34780389

[20] Lieber J Sommerfeldt DW. [Diametaphyseal forearm fracture in childhood. Pitfalls and recommendations for treatment]. Unfallchirurg. (2011) 114(4):292–9. 10.1007/s00113-011-1962-5 21445651

[21] Kose O Deniz G Yanik S Gungor M Islam NC. Open intramedullary kirschner wire versus screw and plate fixation for unstable forearm fractures in children. J Orthop Surg (Hong Kong). (2008) 16(2):165–9. 10.1177/230949900801600207 18725665

[22] Di Giacinto S Pica G Stasi A Scialpi L Tomarchio A Galeotti A The challenge of the surgical treatment of paediatric distal radius/ forearm fracture: k wire vs plate fixation—outcomes assessment. Med Glas (Zenica). (2021) 18(1):208–15. 10.17392/1315-21 33336564

[23] Serbin R Duemmler M Bonvillain K Coe K Habet NA Odum S Does sagittal alignment matter? A biomechanical look at pinning pediatric supracondylar humerus fractures. J Pediatr Orthop. (2025) 45(1):16–21. 10.1097/BPO.0000000000002809 39254208

[24] Ozkan S Westenberg RF Helliwell LA Mudgal CS. Distal radius fractures: evaluation of closed reduction and percutaneous Kirschner wire pinning. J Hand Microsurg. (2018) 10(3):134–8. 10.1055/s-0038-1648334 30483019 PMC6255731

[25] Wang C Su Y. Radial and ulnar medullary canal diameter in children: anatomical limitations of elastic stable intramedullary nailing. Front Surg. (2022) 9:882813. 10.3389/fsurg.2022.882813 36386535 PMC9649893

